# Detection and molecular characterization of carbapenem-resistant gram-negative bacterial isolates

**DOI:** 10.1186/s13568-024-01780-7

**Published:** 2024-11-13

**Authors:** Aliaa A. Mustafa, Hala Abushady, Reda Roshdy, Sawsan Y. Elateek, Ayman K. El Essawy

**Affiliations:** 1 Department of Microbiology, Faculty of Science, Ain Shams University, Cairo, Egypt; 2 Department of Microbiology, National Institute of Hepatology, Cairo, Egypt; 3 Department of Genetics, Faculty of Agriculture, Ain Shams University, Cairo, Egypt; 4 Department of Microbiology, Specialized Hospital, Ain Shams University, Cairo, Egypt

**Keywords:** Carbapenem-resistant bacteria (CRB), Gram-negative bacteria (GNB), Chromogenic medium, 16SrRNA, VITEK 2, CRGNB

## Abstract

Antimicrobial-resistant bacteria (ARB) are responsible for increased mortality and morbidity. Therefore, this study focuses on evaluating traditional and molecular diagnostic tools of carbapenem-resistant gram-negative bacteria (CRGNB). In order to achieve this, 94 samples, from different patients’ specimens, and surrounding environment, were collected from intensive care units (ICUs) at Ain Shams University Specialized Hospital and the National Cancer Institute, Cairo, Egypt. The swabs were cultured on appropriate media, including Chromogenic medium (HiCrome KPC Agar Base “HIMEDI AM1831”), and MacConkey-10 µg imipenem disc resulting in 136 isolates with different culture characteristics. Next, the selected isolates were subjected to VITEK 2 machine and 16SrRNA (16 S ribosomal RNA) sequencing. The sensitivity of HiCROME KPC agar for CRGNB detection was 99.3% and 94.7%, in reference to the MacConkey-disc and VITEK-2 methods, respectively. The HiCrome KPC agar assumptions for bacterial identification were not as consistent as those of VITEK 2 (with only 47.4% agreement) and 16SrRNA gene sequencing analysis. The approaches discussed in this study facilitate providing rapid diagnosis and treatment of CRGNB, which helps increase survival rates. HiCrome KPC agar is considered a relatively accurate and easy method that can be used in any laboratory. In addition, the selected strains were deposited in the gene bank with the accession numbers OR553657, OR553658, and OR553659. It is noteworthy that Genus *Acinetobacter* is the major CRGNB isolated from the patients and environmental surfaces in the hospitals. This highlights the importance of proper environmental and terminal cleaning procedures in healthcare facilities and applying control measures to ensure infection prevention.

## Introduction

Antimicrobial resistance is a global public health concern that mostly results from poor hygiene, abuse of antibiotics, and delayed administration of adequate antibiotics (Katiyar et al. 2020). The global spread of antimicrobial-resistant organisms (ARO) has been identified recently by the World Health Organization (WHO), the European Union, the US Government, and the Centers for Disease Control and Prevention (USA) as one of the most significant threats to human health (Seale et al. 2017). Medical practitioners consider CRGNB a hazard since the infections induced by such bacteria are responsible for increased mortality and morbidity among patients (Mahapatra et al. 2021). CRGNB exhibits high resistance not only to β-lactam antimicrobials but also to most other classes of antibiotics (Kakoullis et al. 2021). Furthermore, CRGNB are multidrug-resistant due to the existence of carbapenemases, which are hydrolyzing enzymes that break down most β-lactam antibiotics (Aurilio et al. 2022). Carbapenems are bactericidal β-lactam antimicrobials that are efficient in controlling serious infections induced by extended-spectrum β-Lactamase (ESBL)-producing bacteria (Codjoe and Donkor 2018). Additionally, they have a wide range of antibacterial action and a specific structure characterized by a carbapenem coupled to a β-lactam ring which confers protection against most β-lactamases (Codjoe and Donkor 2018). This shows that the carbapenem resistance mechanism is based on the carbapenem hydrolyzing action of the carbapenemase enzyme (Nordmann and Poirel 2019).

In addition, β-lactams are almost hydrolyzed by β-lactamases (Kapoor et al. 2017). β-lactamase enzymes are categorized into 4 groups (i.e., A, B, C, and D) based on their central catalytic domain and substrate preference. Carbapenemases are included in classes A, B, and D. On the other hand, class C enzymes typically hydrolyze cephalosporins. Class A, C, and D enzymes possess serine at the active catalytic site, while class B enzymes contain Metallo-β-lactamases (MBLs) and zinc at the active site (Nordmann and Poirel 2019). In fact, several tests are available for rapid detection of carbapenem resistance. They can be divided into two categories: molecular tests, which detect the resistance mechanism (i.e. presence of a carbapenemase gene); and novel, phenotypic tests, which identify the in vitro activity of carbapenemase enzymes (i.e. hydrolysis of carbapenems in vitro) (Banerjeea and Humphries 2017). These methods are used for carbapenemase detection in clinical samples. They include automated systems or disc diffusion, selective agar, spectrometric, 16SrRNA sequencing, and molecular methods (Codjoe and Donkor 2018). It is necessary to recognize the most convenient methods for rapid and accurate diagnosis of such mortal bacteria (Zhang et al. 2022).

Thus, this study aims to identify the rapid and accurate approaches that can be adopted in microbiological laboratories to affirm CRGNB infection. Agar screening assays were performed for detecting CRGNB and were confirmed by VITEK 2 and 16 S rRNA sequencing. Moreover, MacConkey agar plates, Chromogenic medium, and MacConkey agar plates supplemented with imipenem discs were utilized for CRGNB detection; and, then, confirmatory tools such as VITEK 2 (Pan et al. 2018) and 16SrRNA sequencing (Peker et al. 2019) were used for validation.

The study was ethically cleared by the Faculty of Science (Ain Shams University) research ethics committee [ASU-SCI/MICR/2023/6/1].

## Materials and methods

### Samples collection

A total of 94 samples were collected from patients admitted to the intensive care units (ICUs) at Ain Shams University Specialized Hospital and the National Cancer Institute (Cairo, Egypt). They included 46 swabs from the patients’ blood, pus, sputum, mucous, pus, stool, and urine. The patients were of both genders, of different ages, and with different types of infections and diseases. Moreover, the sample assortment was not exclusively limited to patients but also extended to include the surrounding surfaces since 43 swabs were gathered from pillows, floors, beds, walls, and bathrooms in some patients’ rooms and biohazard disposal of the hospitals. On the other hand, the source of the remaining five hospital samples was unknown. Sterile swabs were also used in sampling and refrigerated at 2°–8 °C for 24 h, as recommended (Detection and enumeration of bacteria in swabs and other environmental samples, 2017) until cultivation.

### Screening with traditional methods

All samples were cultured on a Tryptic Soy medium (Oxoid, UK), MacConkey agar(Oxoid, UK), and Chromogenic medium (HiCrome KPC Agar Base - HIMEDI AM1831). Moreover, a sensitivity test took place using MacConkey media with 10 µg imipenem disks (Oxoid, UK) for CRGNB detection. The tested swabs were streaked on HiCrome KPC plates and incubated at 37 °C for 24 h. Each sample was streaked on MacConkey agar plates supplemented with imipenem disc 10 µg. Then, the plates were incubated at 37 °C for 24 h (Ajao et al. 2011). Finally, 19 isolates that represented different sources were tested using VITEK 2.

### Screening with molecular methods

#### 16SrRNA sequencing

Three out of the 19 isolates, which had undergone VITEK 2, were analyzed by 16SrRNA sequencing. These three isolates represented different bacterial genera, based on the results obtained from VITEK 2. In addition, DNA extraction from bacterial isolates was performed using the GeneJET Genomic DNA Purification Kit (K0721). Then, the extracted DNA was sent to Macrogen, Korea where the DNA analyzer was used with the universal primer pairs (Osama et al. 2019); the forward primer sequence was 5`-AGAGTTTGATCATGGCTCAG-3,` and the reverse primer sequence was 5`-ACGAGCTGACGACAGCCATG-3`. Next, the amplicons (DNA) were sent for partial sequencing and analyzed by Applied Biosystems 3730xl (Thermofisher, USA). After that, the partially sequenced 16SrRNA isolates were Chromatogram viewed using the Codoncode Aligner program (Version 4, Codoncode corporation, Dadham, USA), and the contigs were generated. Subsequently, the contigs were blasted on the NCBI genebank database to determine the species using MEGA version 5 (Tamura et al. 2011); and multiple sequence alignments were performed using ClustalW (Thompson et al. 1994). Finally, the phylogenetic trees were generated using the Neighbor-Joining algorithm with 1000 bootstraps (Saitou and Nei 1987).

It is noteworthy to be pointed out that the contigs (1, 2, and 3) produced from 16SrRNA sequencing were 995, 894, and 958 bp, respectively. Moreover, they were deposited in the gene bank with the accession numbers OR553657, OR553658, and OR553659, respectively.

## Results

**Fig. 1:**
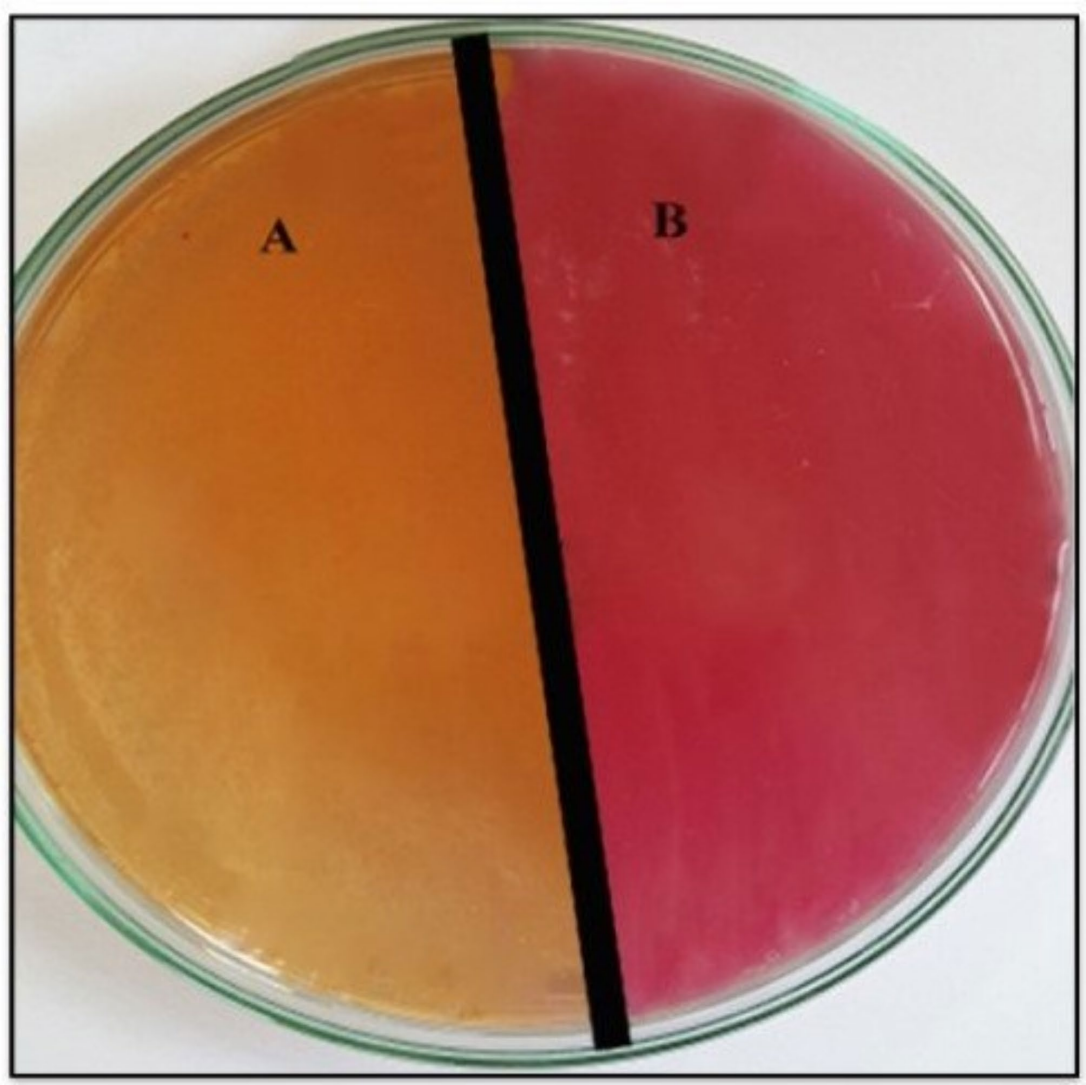
**A** Lactose-fermenter bacteria and **B** non-lactose fermenter bacteria on MacConkey

**Fig. 2:**
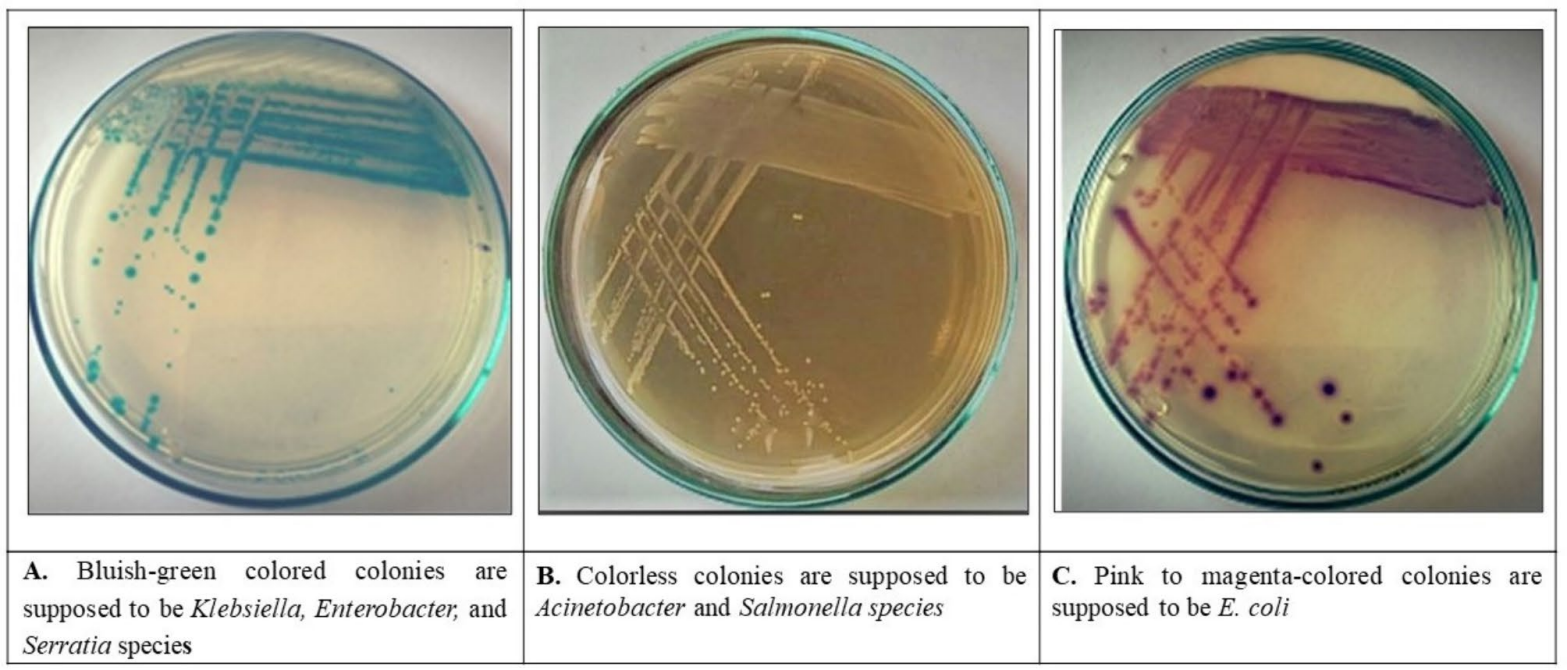
Different types of bacteria of CRGNB on HiCrome KPC agar medium

The 94 samples were screened on MacConkey agar and HiCROME KPC agar media. Out of these samples, 24 showed mixed growth of different types of bacteria for the same swab. These types appeared with different characteristics on MacConkey agar plates as lactose and non-lactose fermenters (Fig. 1). Different colored colonies appeared on the HICROME KPC medium, according to the manufacturer’s instructions (HiMEDIA HiCrome™ KPC Agar Base M1831). The colonies of different colors on the chrome ager referred to different types of bacteria according to the key of HiCROME KPC agar medium, which revealed different enzymatic properties of each strain (Fig. 2). The bluish-green colonies are supposed to be carbapenem-resistant strains of *Klebsiella*,* Enterobacter*, and *Serratia* species (Fig. 2A). Furthermore, the colorless colonies are assumed to be *Acinetobacter* and *Salmonella species* (Fig. 2B). The pink to magenta-colored colonies are supposed to be *E. coli* (Fig. 2C). On the other hand, 10 out of the 94 samples showed no growth on MacConkey agar plates, while their growth appeared clearly on HiCROME KPC agar medium.

**Fig. 3:**
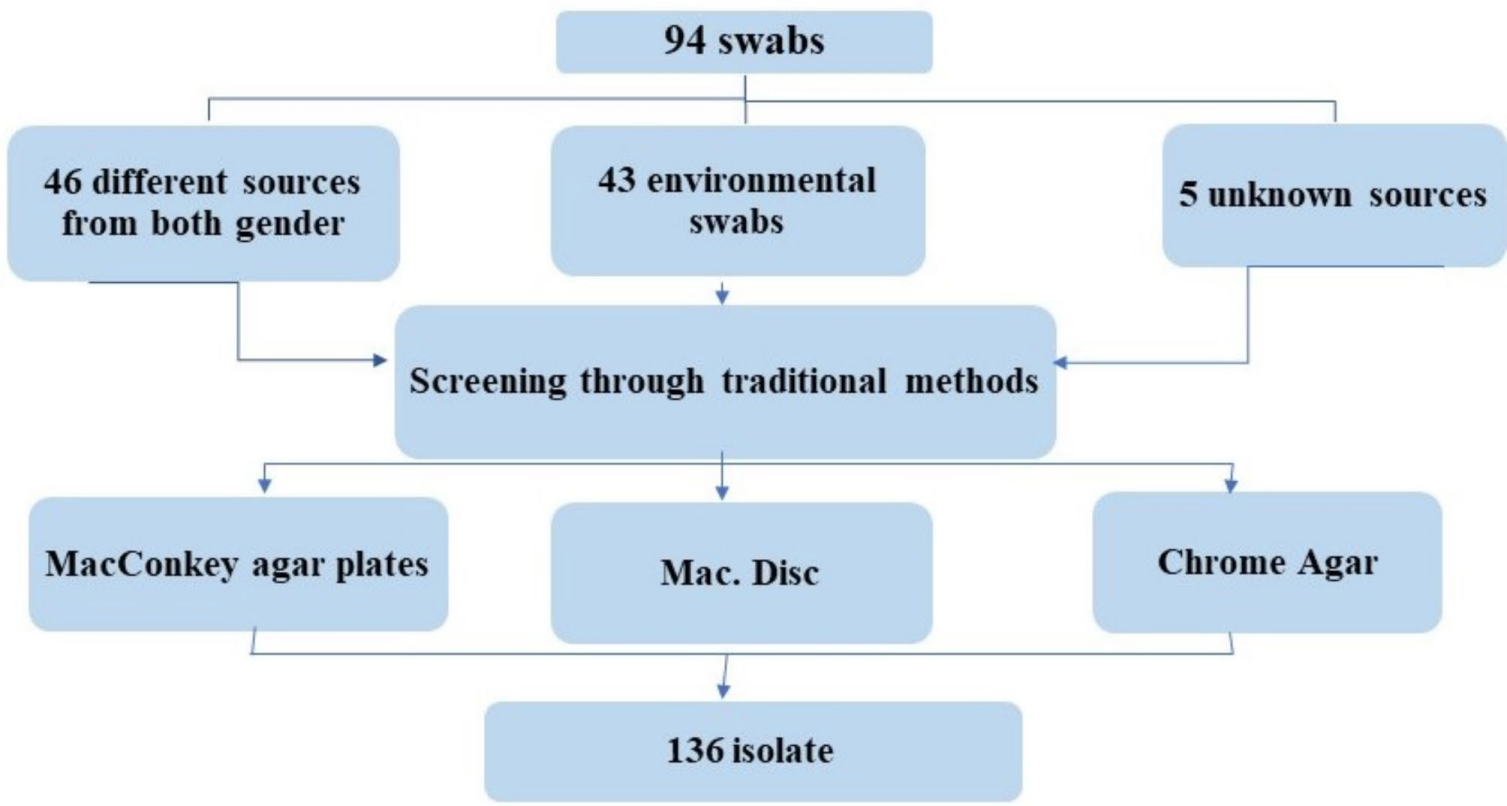
Number of isolated bacteria found using traditional screening methods

The 94 samples provided 136 isolates that appeared with different characteristics on MacConkey agar plates. Different colony colors were detected when applying HiCrome KPC agar plates and sensitivity test (Fig. 3).

The accuracy of HiCrome KPC agar was evaluated in terms of sensitivity and Positive Predictive Value (PPV). All the 136 isolates were analyzed using the Mac. Disc plates (sensitivity test), while 19 isolates were tested using VITEK 2. It was found that the sensitivity of HiCrome KPC agar to detect CRGNB was 99.3% and 94.7, using Mac. Disc and VITEK 2, respectively (Tables 1 and 2).

**Table 1:** Evaluation of HiCrome KPC agar to detect CRGNB adopting the Mac. Disc. Method

	Positive Mac. Disc	Negative Mac. Disc	Total	Sensitivity	Specificity	PPV	NPV
CRGNB positive using HiCrome KPC agar	135	NA	135	99.3%	NA	100%	NA
CRGNB negative using HiCrome KPC agar	1	NA	1				
Total	136	0	136				

PPV, Positive Predictive Value; NPV, Negative Predictive Value

**Table 2:** Evaluation of HiCrome KPC agar to detect CRGNB adopting the VITEK 2 method

	Positive VITEK 2	Negative VITEK 2	Total	Sensitivity	Specificity	PPV	NPV
CRGNB positive using HiCrome KPC agar	18	NA	18	94.7%	NA	100%	NA
CRGNB negative using HiCrome KPC agar	1	NA	1				
Total	19	0	19				

PPV, Positive Predictive Value; NPV, Negative Predictive Value

On the other hand, HiCrome KPC agar was evaluated regarding predicting the isolate type (Presumptive bacterial identification) using VITEK 2. In fact, 10 out of the 19 isolates, that underwent VITEK 2, reflected false predictions. In contrast, nine isolates were predicted correctly by HiCrome KPC agar, which indicates that the correct presumptive identification was just 47.4% (Table 3).

**Table 3:** Evaluation of HiCrome KPC agar to predict isolate type adopting the VITEK 2 method

	VITEK 2 identification of isolates	% of Correct prediction of isolate type using HiCrome KPC agar
Correct prediction of isolate type using HiCrome KPC agar	9	47.4%
False prediction of isolate type using HiCrome KPC agar	10	
Total	19	

**Fig. 4:**
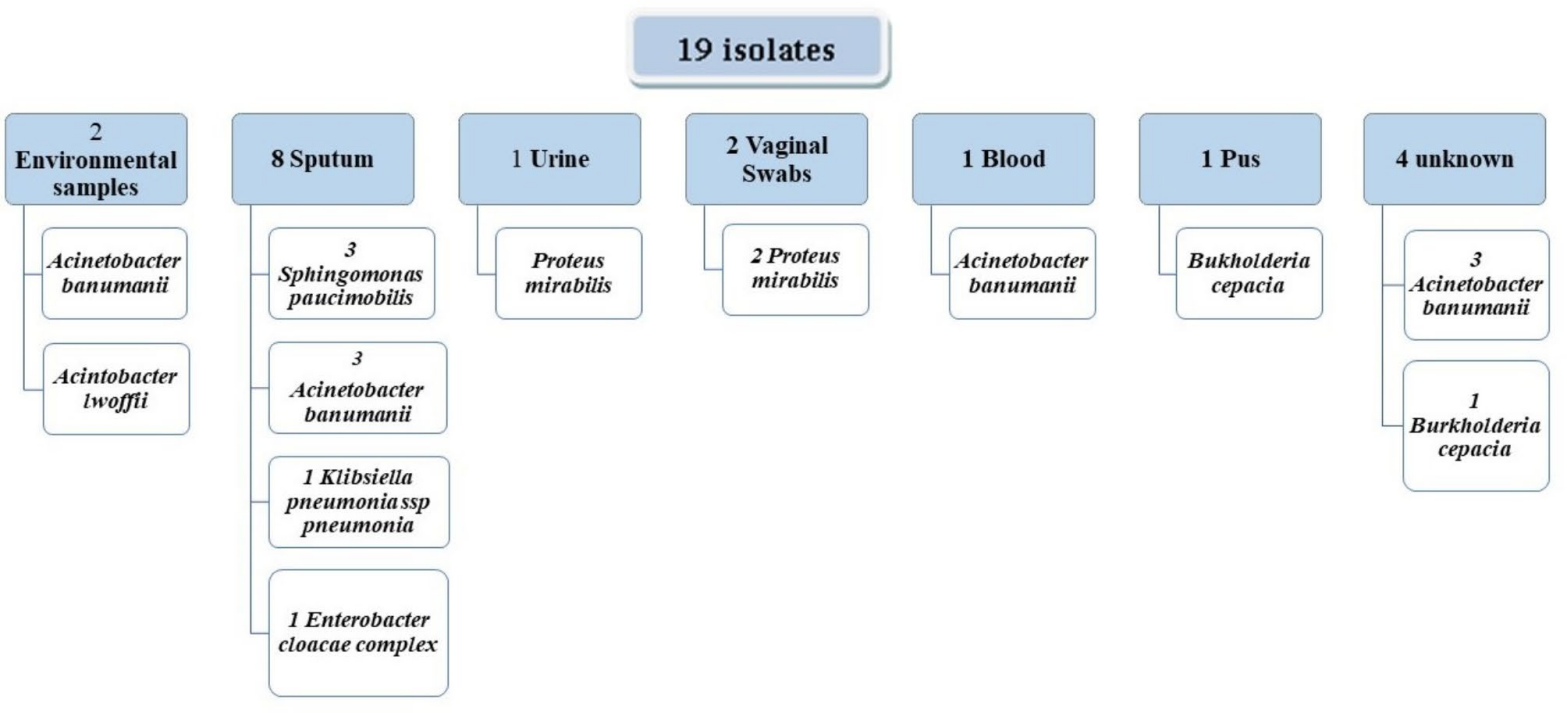
Bacterial isolates undergone VITEK 2 analyses

Out of the 136 isolates, 19 were analyzed using VITEK 2 (Fig. 4). They were selected as illustrative representatives of each sampling source. Two of these 19 isolates were environmental samples collected from the patients’ surroundings, while the rest were patients’ specimens, such as sputum, Urine, and blood.

These two environmental isolates were *Acinetobacter banumanii* and *Acinetobacter lwoffii*, while the other isolates from the patients’ specimens were *Bukholderia cepacian*,* Enterobacter cloacae complex*,* Sphingomonas paucimobilis*,* Klebsiella pneumonia*,* Proteus mirabilis*,* and Acinetobacter banumanii.* All these isolates analyzed using VITEK 2 were CRGNB since they grew on MacConkey, MacConkey with 10 µg imipenem disc, and HiCrome medium.

**Fig. 5:**
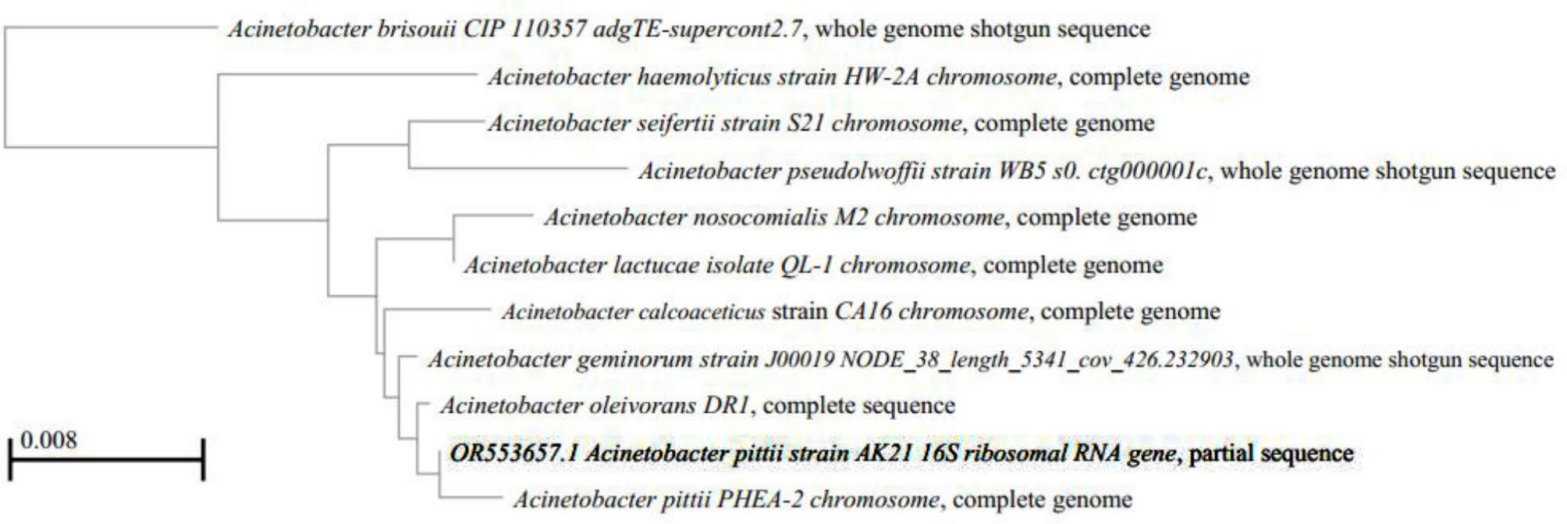
The phylogenetic tree of isolate #1 (Acinetobacter pittii) was generated using the neighbor-joining method with 1000 bootstraps

**Fig. 6:**
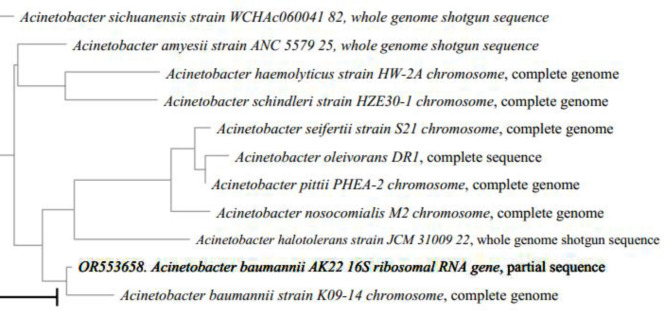
The phylogenetic tree of isolate #2 (Acinetobacter banumanii) was generated using the neighbor-joining method with 1000 bootstraps

**Fig. 7:**
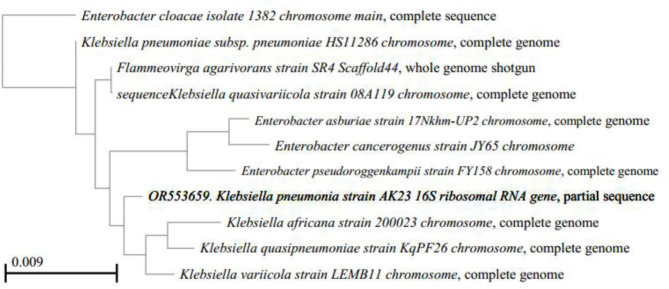
The phylogenetic tree of isolate #3 (Klebsiella pneumoniae) was generated using the neighbor-joining method with 1000 bootstraps

Moreover, partial 16SrRNA sequencing was used to identify three isolates assigned from the 19 isolates identified by VITEK 2. Isolates 1, 2, and 3 were partially sequenced using 16SrRNA universal primers. The contigs (1, 2, and 3) produced 995, 894, and 958 bp, respectively. Furthermore, they were deposited in the gene bank with the accession numbers OR553657, OR553658, and OR553659, respectively. Subsequently, the nearest species matched with the isolate sequences were imported from the gene bank and were added to the query to perform multiple sequence alignments with ClustalW and computing pairwise distances, with maximum composite likelihood. Additionally, phylogenetic trees were generated using the Neighbor-joining method with 1000 bootstraps (Figs. 5, 6 and 7).

## Discussion

The increasing frequency of CRGNB has been linked to the widespread usage of carbapenems in hospitals, especially when infection control practices are below normal levels (Bratu et al. 2005; Alotaibi et al. 2017). The prevalence of CRGNB in some regions of the world highlights the demand for adequate methods to detect these types of bacteria and prevent the dissemination of CRGNB. Obviously, this is essential to identify patients at risk of invasive infection, report isolates with carbapenemase activity as soon as possible, and use proper infection control measures (Alizadeh et al. 2018).

The result obtained regarding the non-growth of the ten samples applying MacConkey was attributed to the low aggregation of the bacterial population in those samples, which hindered the growth of bacteria; however, growth was detected by HiChrome KPC medium (Mahapatra et al. 2021). This finding highlights an additional advantage of the HiChrome KPC medium.

Moreover, the high percentage of imipenem resistance may be due to the excessive admission of imipenem with other antibiotics. This is agreeable with the study by Abda et al. (2020) who reported high resistance to imipenem in the bacterial populations they screened; they detected 79 imipenem-resistant bacteria among isolates from 153 patients that belonged to eight genera with clinically relevant microorganisms. Additionally, Meyer et al. (2010) conducted a study in Germany and reported that carbapenem-resistant pathogens increased twofold with carbapenem treatment. These studies also indicated that the disc carbapenem test is affordable and works quickly for CRGNB diagnosis.

In recent years, chromogenic agar media has been recognized as a successful method for detecting CRGNB (Alizadeh et al. 2018; Bourles et al. 2023), especially when the infections belong to the *Enterobacteriaceae* family (Memar et al. 2017; van Almsick et al. 2018) that causes most of the infections in hospitals and health care institutions due to its resistance to β- lactams antimicrobial agents (Gautier et al. 2018).

In our study, HiCrome agar showed 99.3% and 94.7% sensitivity regarding the detection of CRGNB, when adopting Mac. Disc and VITEK 2, respectively. This is considered acceptable in comparison to the study of Mahapatra et al. (2021) in which HiCrome sensitivity was 100% regarding screening CRGNB. Nevertheless, Mahapatra et al. (2021) revealed that the sensitivity of the chromogenic medium for a certain type of carbapenemase (OXA-48 producers) was only 43%. On the other hand, the sensitivity of HiCrome KPC in our study was 47.4% regarding detecting the type of bacteria, based on VITEK 2 identification. This result indicates that HiChrome agar is an effective and rapid method for CRGNB detection; however, it is not a reliable method for proper identification. Despite this, the early detection of carbapenem resistance based on this method will significantly help clinicians as well as infection control practitioners to treat patients and control the spread of this multi-drug-resistant bacteria.

In addition, it was intriguing that the environmental sample isolates were *Acinetobacter*, while the patient’s specimens had multiple types of CRGNB that included 7 *Acinetobacters*. This confirms that the lack of adequate hygiene can be as lethal as the resistance to antibiotics; and leads to mortality and morbidity in hospitals and health care institutions. *Acinetobacter* can result in hospital-acquired infections; moreover, it can acquire antibiotic resistance, especially carbapenems (Cheng et al. 2015). Since *Acinetobacter* can spread through equipment, tools, water, food, hands, and even infected patients, it causes major threats worldwide (Bradford et al. 2001) in the most vulnerable places to infection, such as hospitals (Gautier et al. 2018; Alizadeh et al. 2018).

Isolate 1 was extracted from an environmental swab sample from the area surrounding the patient, while isolate 2 was obtained from a human specimen (infected patient). Both were found to be *Acinetobacters*, which confirms the spread of such antibiotic-resistant bacteria throughout hospitals and community-acquired infections (Howard et al. 2012; Nguyen and Joshi 2021). Furthermore, the three isolates identified using VITEK 2 were *Acinetobacter baumannii*,* Burkholderia cepacia*, and *Klebsiella pneumonia.* However, when it came to identifying them via 16SrRNA sequencing, two of them matched the VITEK 2 identification results. To illustrate, isolate 1 was identified as *Acinetobacter pittii* by 16SrRNA but as *Acinetobacter* baumannii by VITEK 2. Isolate 2 was identified by 16SrRNA as *Acinetobacter banumannii*, while by VITEK 2 as *Burkholderia*; the two identifications were related at the phyllum level of Pseudomonadota. Isolate 1 shared 99.8% similarity with *Acinetobacter pittii* str. NC_016603 with E-value = 0, while isolate 2 shared 99.44%; similarity with *Acinetobacter banumannii* 016901DP_1, 016901DP_2, and 1,007,214 with E-value = 0. We could not genetically confirm that isolate 1 as an environmental isolate of *Acinetobacter* is the same specific human isolate from the hospital, but the genus *Acinetobacter* is the major genus isolated from human sources in the current study.

Moreover, the identification of isolate 3 by 16SrRNA matched with VITEK 2 results as being *Klebsiella pneumonia.* It grew on both Chrome agar and MacConkey during the screening step for CRGNB (Ajao et al. 2011; Tejashree and Hegde 2017). Then it underwent VITEK 2 and 16SrRNA analysis for the identification of bacterial isolates. It shared 99.58% partial 16SrRNA gene sequencing similarity with *Klebsiella pneumoniae*. It belongs to the *Family Enterobacteriaceae* and *Genus Klebsiella; i*t showed similarity to *Klebsiella pneumoniae* NC_016845, with E- value = 0. This isolate was cultivated from the sputum of an infected patient who was admitted to the hospital with previous different bacterial infections. However, our diagnostic methods confirmed the infection with CRGNB, which proves that there can be mixed infections in healthcare facilities (Cheng et al. 2022). Mixed infections may be due to several significant risk factors such as mechanical ventilation, excessive antibiotic exposure, anti-microbial admission, and organ transplantation (Hussein et al. 2009; Alotaibi et al. 2017).

The rising prevalence of CRGNB in some regions of the world highlights the demand for a sensitive screening method to detect these bacteria and stop the spread of CRGNB. This is essential to rapidly report isolates with carbapenemase activity, apply the proper infection control measures, and identify patients who are colonized and at risk of invasive infection. For the quick identification of CRE, PCR-based approaches are extremely sensitive and reliable. Nevertheless, many centers lack the necessary skills and budget to apply these methods. Hence, we conclude that the HiCrome KPC medium method is a relatively accurate, rapid, cheap, and easy approach to achieve this.

Additionally, the major CRGNB participant in our study was the genus *Acinetobacter*, followed by other genera including *Burkholderia*,* Proteus*,* Klebsiella*,* Enterobacter*, and *Sphingomons*. The only confirmed genus isolated from the hospital’s environmental surfaces was *Acinetobacter*. Accordingly, we stressed the importance of proper routine environmental and terminal cleaning in healthcare facilities, with special attention to isolation rooms and wards where infected patients are admitted.

## Data Availability

The authors can provide the data of this study upon reasonable request.

## Abbreviations

ARB: Antimicrobial-resistant bacteria
CRGNB: Carbapenem-resistant gram-negative bacteria
ICUs: Intensive care units
CRB: Carbapenem-resistant bacteria
GNB: Gram-negative bacteria
ARO: antimicrobial-resistant organisms
WHO: World Health Organization
MBLs: Metallo-β-lactamases
PPV: Positive predictive value
NPV: Negative predictive value
